# LDHA: The Obstacle to T cell responses against tumor

**DOI:** 10.3389/fonc.2022.1036477

**Published:** 2022-11-28

**Authors:** Yu Tang, Shuangshuang Gu, Liqun Zhu, Yujiao Wu, Wei Zhang, Chuanxiang Zhao

**Affiliations:** ^1^ Department of Gastroenterology, Affiliated Hospital of Jiangsu University, Zhenjiang, Jiangsu, China; ^2^ Shanghai Institute of Rheumatology, Shanghai Renji Hospital, Shanghai Jiaotong University School of Medicine, Shanghai, China; ^3^ Institute of Medical Genetics and Reproductive Immunity, School of Medical Science and Laboratory Medicine, Jiangsu College of Nursing, Huai’an, Jiangsu, China

**Keywords:** LDHA, lactate, T cell responses, tumor, metabolic reprogramming

## Abstract

Immunotherapy has become a successful therapeutic strategy in certain solid tumors and hematological malignancies. However, this efficacy of immunotherapy is impeded by limited success rates. Cellular metabolic reprogramming determines the functionality and viability in both cancer cells and immune cells. Extensive research has unraveled that the limited success of immunotherapy is related to immune evasive metabolic reprogramming in tumor cells and immune cells. As an enzyme that catalyzes the final step of glycolysis, lactate dehydrogenase A (LDHA) has become a major focus of research. Here, we have addressed the structure, localization, and biological features of LDHA. Furthermore, we have discussed the various aspects of epigenetic regulation of LDHA expression, such as histone modification, DNA methylation, N6-methyladenosine (m^6^A) RNA methylation, and transcriptional control by noncoding RNA. With a focus on the extrinsic (tumor cells) and intrinsic (T cells) functions of LDHA in T-cell responses against tumors, in this article, we have reviewed the current status of LDHA inhibitors and their combination with T cell-mediated immunotherapies and postulated different strategies for future therapeutic regimens.

## Introduction

Nowadays, a tumor still represents a grave life threat to humanity and has become the leading cause of mortality. The conventional regimens for tumors still rest on surgery, radiotherapy, and chemotherapy, whereas the curative efficiency and efficacy have not been satisfactory (1). Hopefully, immunotherapy, as a new generation of tumor therapy, aims to challenge or mobilize the immune system to control and destroy tumor cells (2). T cells are a category of crucial components of the immune system, with activated T cells mediating the engagement of the immune system in the elimination of malignant tumor cells. Over the past decades, T cell-mediated immunotherapies, such as immune checkpoint blockade (ICB) therapy and adoptive T-cell therapy (ACT), have gained considerable therapeutic successes in a certain range of solid tumors and hematological malignancies, which promise the dawn for a complete remission of tumors (3). Unfortunately, this immunotherapeutic efficacy is frequently hindered in many other solid tumors. In addition, a wealth of studies have revealed that the immunotherapeutic inefficiency is implicated with cellular metabolic reprogramming of tumor cells and T cells (4).

Cellular metabolic reprogramming determines the functionality and viability of both cancer cells and T cells (5). Metabolic reprogramming, particularly glucose catabolism, is a hallmark of tumors (6). Tumor cells represent a transition in glucose utilization from mitochondrial oxidative phosphorylation to glycolysis, even in the presence of oxygen, to form lactate and ATP, a process known as aerobic glycolysis or the “Warburg effect” (7). This metabolic rewiring commonly results in a nutrient-depleted, acidic, and hypoxic immunosuppressive tumor microenvironment (TME). With respect to T cells, metabolic reprogramming is related to their activation and differentiation. Generally, naive T cells have a low glycolytic level and mainly rely on mitochondrial oxidation of fatty acids (FAO) for energy during the quiescence state. On activation, effector T cells switch to aerobic glycolysis or simultaneously upregulate oxidative phosphorylation to meet the energy and anabolic demands while inhibiting FAO (8). Thus, aerobic glycolysis exerts profound impacts on T cell-mediated antitumor immunity in the TME, as illustrated in the increased glycolytic metabolism in melanoma cells, which explicates the resistance to ACT and ICB (9, 10).

One of the key enzymes involved in glycolysis is lactate dehydrogenase A (LDHA), the catalyst of the conversion of pyruvate to lactate with the oxidation of nicotinamide adenine dinucleotide dehydrogenase (NADH) to NAD^+^ (11). Current knowledge has established that LDHA is involved in tumor initiation, development, progression, invasion, metastasis, angiogenesis, and immune escape (12). Additionally, LDHA functions as a biomarker for tumor diagnosis and prognosis (11, 12). Accordingly, LDHA has become an attractive target for possible pharmacological approaches in cancer therapy. In this review, we illustrated the LDHA structure, location, and biological features as well as the epigenetic mechanisms of LDHA expression. With a focus on the extrinsic (tumor cells) and intrinsic (T cells) effects of LDHA on T-cell responses against tumors, we reviewed the prevailing studies on LDHA-targeted therapies in order to address the prospect of LDHA inhibitors combined with T cell-mediated immunotherapy as a therapeutic strategy.

## The structure, cellular localization, and biological features of LDHA

**Figure 1:**
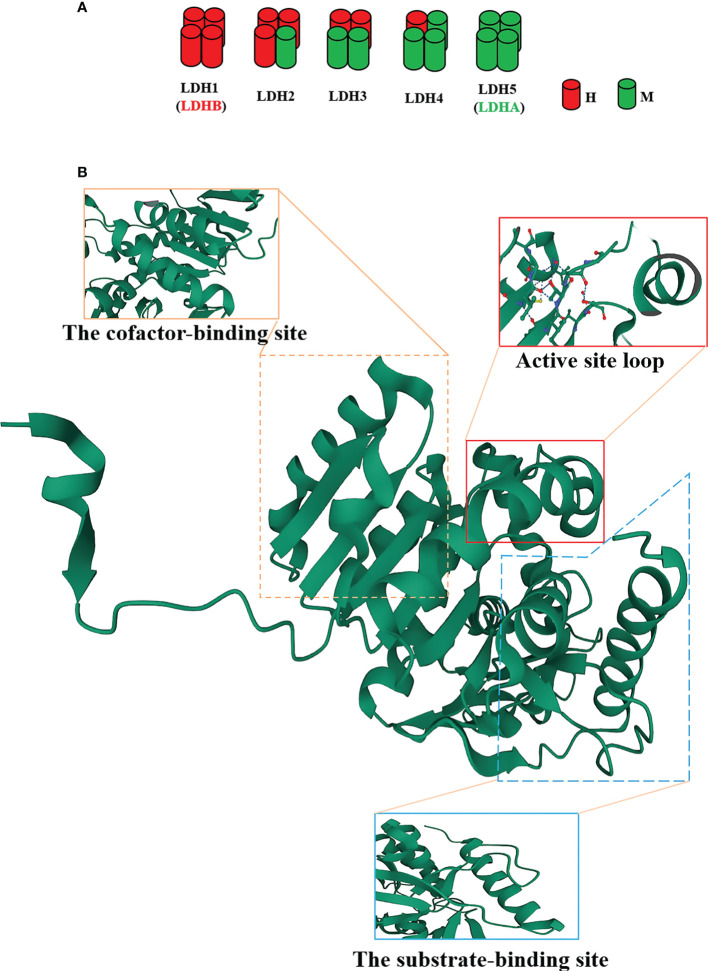
The structure of the lactate dehydrogenase A (LDHA) subunit. **(A)** LDH isoenzymes are LDH-1 (4H), LDH-2 (3H1M), LDH-3 (2H2M), LDH-4 (1H3M), and LDH-5 (4M). **(B)** Tertiary structure of an LDHA subunit. LDHA contains 332 amino acids. The N-terminal region possesses an unstructured region formed by 20 amino acids to interact with the C-terminus of another adjacent subunit. Residues 99-110 form the conformation of a flexible “active site loop,” and Arg105 is responsible for the trapping of adhered pyruvate. Residues 20-162 and 248-266 constitute the cofactor-binding site, which is characteristic of three parallel β-strands enclosing two α-helices. Residues 163-247 and 267-331 comprise the mixed α/β substrate-binding domain.

LDHA is a protein with 332 amino acids, which is encoded by LDHA genes with eight exons located on chromosome 11p15.1 (13). As well acknowledged, LDHA is a constituent subunit (M) of LDH in combination with LDHB subunit (H) to form five active LDH isoenzymes (**Figure 1A**), i.e., LDH-1 (4H), LDH-2 (3H1M), LDH-3 (2H2M), LDH-4 (1H3M), and LDH-5 (4M) (13). Of note, LDH1 and LDH5 are commonly known as LDHB and LDHA, respectively. LDHA is favored in low-oxygen tissues and is more effective in catalyzing pyruvate to lactate; conversely, LDHB prefers to exist in tissues with a potent aerobic metabolism and preferentially converts pyruvate to acetyl coenzyme A for entry into the tricarboxylic acid cycle (TAC) (14). As validated in a number of studies, the LDHA expression is upregulated in cancer cells (12, 15) in contrast to the approximately intact expression levels of LDHB in normal and carcinomatous tissues (12).

The structure of LDHA subunit has been unfolded (**Figure 1B**). Its N-terminus possesses a haphazard region formed by 20 amino acids to interact with the C-terminus of an adjacent subunit, a critical clue to the formation of LDH (16, 17). The residues 99-110 form the conformation of a flexible “active site loop,” which is often referred to as the substrate specificity loop and contributes to the LDHA catalysis (18). The well-preserved Arg105 in this loop is responsible for the trapping of adhered pyruvate (19, 20) *via* contact with nucleotides and substrates for stabilization (21). In addition, the residues 20-162 and 248-266 constitute the larger Rossmann domain, which is characteristic of three parallel β-strands enclosing two α-helices, i.e., the cofactor-binding site (22). At this site, NADH cofactors chiefly bind to four residues (Asp168, Arg171, and Thr246 and the catalytic His195) located in a groove of the central β-sheet (22–25). These residues are significantly involved in the catalytic activity of LDHA owing to the assembly of the geometry of the catalytic sites (22). The residues 163-247 and 267-331 comprise the mixed α/β substrate-binding domain. The substrates, such as pyruvate, interact with three residues (Arg171 and Thr246 along with Ala236) (19). The active site loop, the cofactor-binding site, and the substrate-binding site compose a certain spatial conformation and jointly contribute to the catalysis of LDHA. Consequently, these sites will become the ideal venue for the performance of the inhibitors.

The efforts revealed that the catalytic reaction followed an ordered event. First, NADH binds to the cofactor-binding site with His195. Thereafter, the substrate pyruvate interacts with the substrate-binding site and Arg105. Finally, the active site loop is enclosed to form a desolvated ternary complex, thereby facilitating the hydride transfer (19, 26). Notably, His195 functions as a proton donor that could transfer a hydride ion from the nicotinamide ring of NADH to the carbonyl C-atom of the pyruvate, ultimately triggering a reaction to complete the oxidation of NADH to NAD^+^ and the release of NAD^+^ and lactate (27, 28).

LDHA is located also in the cytoplasm, mitochondrial matrix, and nucleus (12, 29, 30). In the liver, LDHA is mostly present in the mitochondrial matrix, whereas it is mainly localized in the cytoplasm of cancer cells (31, 32). However, regardless of its presence in the mitochondria or cytoplasm, LDHA is mainly implicated in glycolysis (12, 29). In the nucleus, LDHA is likely involved in DNA duplication and transcription *via* its function as a single-stranded DNA-binding protein (SSB) (30). Another report described that nuclear LDHA induced the production of α-hydroxybutyrate and disruptor of telomeric silencing 1-like (DOT1L)-mediated histone H3K79 hypermethylation in a noncanonical manner of enzyme activity (33). These findings illuminate the avenue to elucidate the novel role of LDHA in the body.

## The epigenetic regulation of the LDHA expression

**Figure 2:**
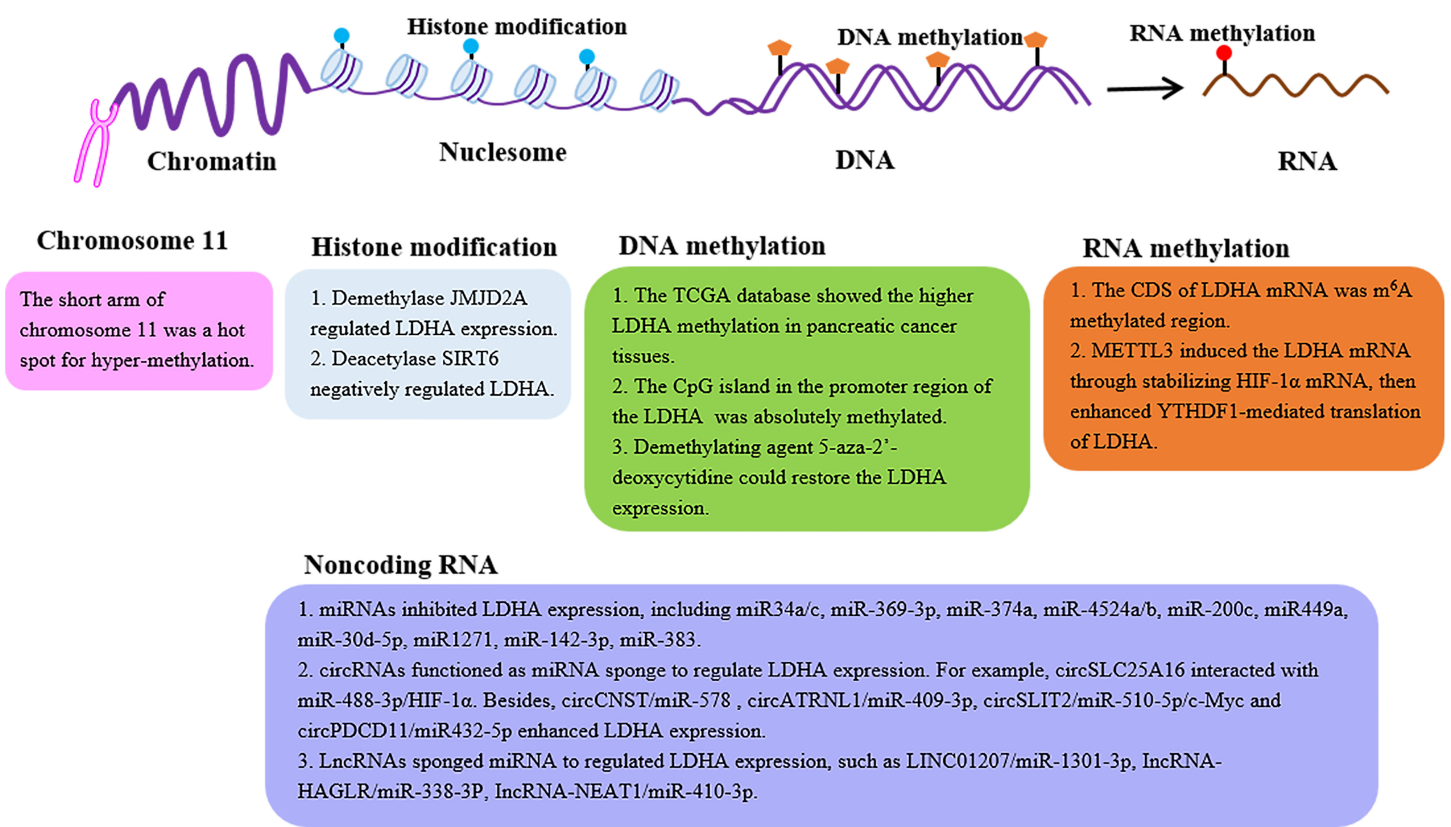
The epigenetic regulation of the LDHA expression. LDH expression can be regulated by epigenetic modifications, such as histone modification, DNA methylation, m^6^A RNA methylation, and noncoding RNA. LDHA, lactate dehydrogenase A; JMJD2A, Jumonji domain-containing protein 2A; SIRT6, sirtuin 6; CDS, coding sequence; METTL3, methyl-transferase-like 3; YTHDF1, YTH domain-containing family protein 1.

With the discovery of LDHA, the mechanisms underlying its expression have been extensively mined. The details of regulatory mechanisms, such as transcription factors and posttranslational modification regulations, have been summarized (34, 35). In the section below, we focused on the LDHA expression profiles from the perspective of epigenetic modifications, such as histone modification, DNA methylation, N6-methyladenosine (m^6^A) RNA methylation, and noncoding RNA (**Figure 2**).

### Histone modification

Histones are the basic structural proteins of eukaryotic chromosomes. The N-terminal amino tail of core histone can undergo posttranslational modifications, including methylation, acetylation, phosphorylation, glycosylation, and ubiquitination, which affect gene transcription (36).

Histone methylation is a modification primarily in arginine and lysine, which is a reversible process regulated by histone methyltransferase (lysine methyltransferase, arginine methyltransferase) and histone demethylase to jointly affect the expression of target genes (36). Jumonji domain-containing protein 2A (JMJD2A) is a histone demethylase. In patients with nasopharyngeal carcinoma (NPC), the JMJD2A level was reported to be positively correlated with the LDHA expression (37), further demonstrating the JMJD2A-regulated LDHA expression at the level of transcription by the combination with the LDHA promoter region (37). Histone acetylation is also a modification dynamically regulated by histone acetyltransferase and histone deacetylase (HDAC), which plays a pivotal role in nucleosomal assembly, structural maintenance of chromatin, and gene transcription (36). Furthermore, histone deacetylase sirtuin 6 (SIRT6) negatively regulates the main glycolytic genes including LDHA (38). In nasal polyp fibroblasts, the decreased expression of SIRT6 resulted in the upregulation of LDH (39).

With respect to the effects of other histone modifications on the LDHA expression, further work is needed.

### DNA methylation

DNA methylation is a process in which the DNA methyltransferase (DNMT) utilizing S-adenosylmethionine (SAM) as the methyl donor transfers the methyl group to the cytosine 5 site of the genomic CpG islands to construct 5-methylcytosine (m5C) (40). In the normal human genome, the CpG islands are in a non-methylated state, whereas the aberrant hypermethylation of certain CpG islands can lead to the corresponding gene silencing (40). LDHA is located on the short arm of chromosome 11, a hot spot for hypermethylation in human tumors (41). Researchers analyzed The Cancer Genome Atlas (TCGA) database and identified the remarkably higher LDHA methylation in pancreatic cancer tissues (42). Furthermore, PCR uncovered that the CpG island in the promoter region of the LDHA gene was indeed methylated (43). There is a similar result reported in the mutant isocitrate dehydrogenase (IDH^mut^) glioma that the LDHA promoter showed increased methylation, leading to its low expression (44). Not surprisingly, the loss of the promoter methylation of LDHA and the higher LDHA expression were evidenced in the IDH^nut^ aggressive glioma (45, 46). Thus, the demethylating agent 5-aza-2′-deoxycytidine could restore the LDHA expression in the retinoblastoma cell line NCC-RbC-51 (43). More importantly, the demethylation of certain CpG sites in the promoter region results in the alteration of LDHA expression contributing to the development of tamoxifen resistance in the breast cancer cell line MCF-7 (47). These findings validate the impact of DNA methylation of LDHA on the patient’s response to chemotherapy in clinical treatment.

### N6-methyladenosine RNA methylation

The m^6^A RNA methylation occurs at the N6-position of adenine in RNA, which regulates RNA splicing, translocation, stability, decay, and translation into proteins (48). The m^6^A modification involves three kinds of crucial protein factors, including methyltransferases (writers), demethylases (erasers), and methylation-binding proteins (readers) (48). To date, only one study revealed that the writer METTL3 enhanced the expression of LDHA (49). Mechanistically, the coding sequence (CDS) of LDHA mRNA is the methylation region for m^6^A, rather than 5′-untranslated region (5′-UTR) or 3′-UTR. Then, METTL3 induced the LDHA transcription *via* the stabilization of the mRNA of hypoxia-inducible factor (HIF)-1α, further enhancing the YTH domain-containing family protein 1 (YTHDF1)-mediated translation of LDHA (49). However, more efforts are needed to elucidate the regulatory effect of the m^6^A modification of LDHA.

### Noncoding RNA

Noncoding RNA refers to RNA that can be transcribed but cannot encode protein, including microRNAs (miRNAs), circular RNAs (circRNAs), and long noncoding RNAs (lncRNAs). Among them, miRNAs with a length of approximately 20–22 nucleotides function as crucial regulators of the gene expression by binding to the 3′-UTR of the target mRNA to inhibit the translation or promote the mRNA degradation (50). Until now, emerging evidence has confirmed the roles for miRNAs in the regulation of LDHA. In colorectal cancer, several miRNAs, such as miR-34a/c, miR-369-3p, miR-374a, and miR-4524a/b, directly bound the 3′-UTR of the mRNA of LDHA to inhibit the LDHA expression (51). Interestingly, there is a point mutation in the 3′-UTR of LDHA (rs18407893 at 11p15.4) in HCT116 colon and BxPC3 pancreatic cancer cells as well as four of 30 samples Aspire of colorectal cancer tissues. This mutation eliminated the binding of miR-374a (51). In addition, miR-200c, miR-449a, miR-30d-5p, miR1271, miR-142-3p, and miR-383 also directly regulated LDHA in different tumors (52–57). Indeed, miR-200c in bladder cancer and miR-449a in non-small-cell lung cancer cell lines were downregulated, enhancing the LDHA level (52, 53). Moreover, several miRNAs (including miR-92-1) indirectly govern the LDHA expression by stabilizing the HIF-1α (58, 59).

circRNAs are another kind of noncoding RNA and are also involved in the regulation of the LDHA expression. A study unveiled that circSLC25A16 interacted with miR-488-3p/HIF-1α to activate LDHA by promoting its transcription in non-small-cell lung cancer (60). Likewise, circ-CNST/miR-578 and circATRNL1/miR-409-3P regulated LHDA in osteosarcoma (61, 62). In pancreatic ductal adenocarcinoma (PDAC), circSLIT2 functioned as a miRNA sponge to target the miR-510-5p/c-Myc axis to activate the transcription by binding to the promoter region of LDHA (63). In addition, circPDCD11 enhanced the LDHA expression by sponging miR-432-5p in triple-negative breast cancer (64). In brief, circRNAs functioned as a miRNA sponge to promote the LDHA expression.

Long noncoding RNAs (lncRNAs) are an important subset of noncoding RNAs, which are the key regulators of gene expression. Recent research authenticated that LINC01207 interacted with miR-1301-3p, the immediate upstream of LDHA (65). In gastric cancer, lncRNA-HAGLR sponged miR-338-3p while LDHA was the direct target of miR-338-3p (66). Additionally, a recent study demonstrated that lncRNA-NEAT1 sponged miR-410-3p to downregulate its expression, thereby inhibiting LDHA in intestinal epithelial cells (IECs) (67).

## The extrinsic (tumor) and intrinsic (T cells) effects of LDHA on T-cell responses to tumors

### The effect of lactate dehydrogenase A in tumor on T-cell responses

**Figure 3:**
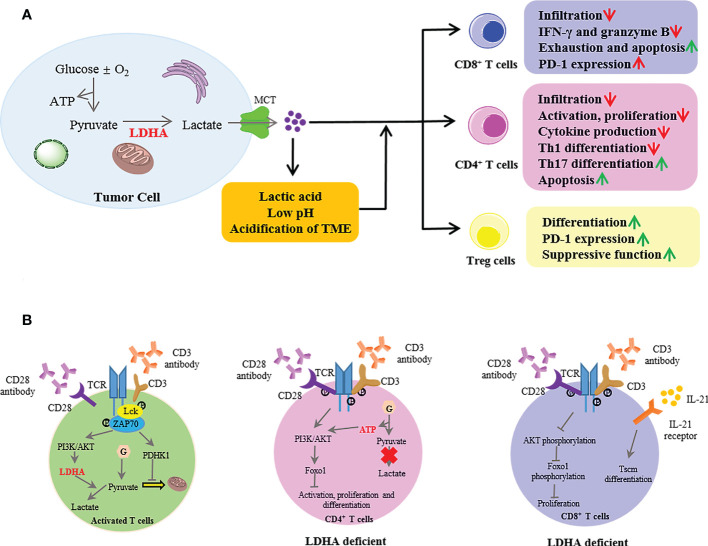
The extrinsic and intrinsic roles of lactate dehydrogenase A (LDHA) in T-cell responses to tumors. **(A)** In the tumor, LDHA catalyzed the conversion of pyruvate to lactate; then, intracellular excessive lactate was excreted from the cytoplasm by MCT into the TME, thus resulting in an extracellular acidic microenvironment with a low pH. The LDHA-lactate-acidic microenvironment established a barrier for T-cell response. MCT, monocarboxylate transporter; TME, tumor microenvironment. **(B)** In activated T cells, when naive T cells were activated with anti-CD3 and anti-CD28, the TCR signaling promoted the activation of PDHK1, suppressing the mitochondrial import of pyruvate. Meanwhile, the TCR induced the LDHA expression through PI3K/AKT signaling in activated T cells, then catalyzed lactate production. LDHA deficiency in CD4^+^ T cells impaired the cell activation and proliferation and the Th17 cell differentiation mediated by the defective termination of the AKT-regulated Foxo1-dependent gene expression program. In CD8^+^ T cells, LDHA deficiency resulted in defective cell expansion *via* impairment of AKT and Foxo1 phosphorylation. Moreover, the LDHA inhibition combined with IL-21 promoted the differentiation into Tscm. PDHK1, pyruvate dehydrogenase kinase 1; PI3K, phosphoinositide 3-kinase; Tscm, T memory stem cells.

Numerous studies have confirmed the elevated LDHA levels in several different cancer types and highly expressed LDHA-mediated tumor immune escape by inhibiting immune killing and promoting immunosuppression (12, 68). In tumor cells, LDHA catalyzes the conversion of pyruvate to lactate, then excessive intracellular lactate is excreted from the cytoplasm by monocarboxylate transporters (MCTs) into the TME, thus resulting in an extracellular acidic microenvironment. Researchers have proposed that the LDHA-lactate-acidic microenvironment can establish a barrier for the T-cell response (**Figure 3A**). The T-cell response is dependent on antitumor effector cells including CD4^+^ and CD8^+^ cells, which orchestrate and perform the antigen-specific killing of cancer cells, respectively. CD4^+^ T cells comprise numerous subsets, such as T helper 1 (Th1) cells that possess a significant antitumor activity and regulatory T (Treg) cells that have an immunosuppressive role and protect tumor cells from other killer cells. CD8^+^ cells are critically important in direct killing of tumor cells *via* the induction of apoptosis and cytokine secretion [interferon (IFN)-γ, granzyme B].

LDHA in tumors negatively affects the immune cell infiltration (9, 69). In a mouse model of melanoma and pancreatic tumor with low or null LDHA, the infiltration and activation of CD8^+^ T cells and NK cells were enhanced, and these infiltration cells produced the increased IFN-γ and granzyme B (69, 70). Similar results have been found in breast tumors that the shRNA-mediated reduction of LDHA enhanced the infiltration of CD3^+^ and CD4^+^ T cells (71). Additionally, LDHA was negatively associated with the T‐cell activation markers (granzyme K and CD25) in human melanoma (69). Moreover, the infiltration of Treg cells was attenuated (70). However, a study reported that renal cell carcinoma (RCC) with a high expression of LDHA showed significant multiplication of T cells (including CD3^+^, CD8^+^, and Foxp3^+^ T cells) and decreased effector molecules (granzyme B and perforin) in these tumor-infiltrating T cells (72), suggesting that RCCs are infiltrated by functionally inactive cytotoxic T cells. These findings indicate that the modulation by LDHA has more effect on the activity than on the population of T cells.

The regulatory effect of LDHA on immune escape by infiltrating T cells is mainly dependent on the excessive lactate secretion from tumor cells to the TME, which might reach levels of up to 10–40 mM over 10 times greater than physiological lactate concentrations (73). High lactate levels could increase Treg cells through a lactate-based nuclear factor (NF)-κB activation and FoxP3 expression as well as drive Foxp3 metabolically reprogrammed T cells to allow Treg cells to work efficiently (74, 75). Furthermore, Treg cells actively absorbed lactate *via* MCT1 and promoted the expression of programmed death 1 by enhancing the nuclear factor of activated T cell (NFAT)-1 translocation into the nucleus (76). Moreover, lactate attenuated the differentiation of the antitumoral Th1 subset by triggering the SIRT1-mediated transcription factor T-bet deacetylation (75), while sodium lactate induced the Th17 differentiation (77). In acute myeloid leukemia (AML), lactate induced the exhaustion of CD8^+^ T cells by altering the lytic granule exocytosis and promoting a higher PD-1 expression (77, 78). However, in mice bearing transplanted MC38 tumors, subcutaneous administration of sodium lactate increased the proportion of stem-like T cell factor-expressing CD8^+^ T cells among intratumoral CD3^+^ cells, and its potential mechanism was mediated by enhancing the acetylation at H3K27 of the Tcf7 super enhancer locus to increase the Tcf7 gene expression (79). Furthermore, lactate anions increased the T cell receptor-dependent cytokine production *via* the glyceraldehyde phosphate dehydrogenase (GAPDH)-mediated posttranscriptional pathway, which promotes the antitumor function *in vivo* (80). Additionally, once the concentration of lactate is above 20 mM, it induced the apoptosis of CD8^+^ T and Natural Killer cells (69).

These above studies only focused on the effect of lactate molecules on T cells without considering that lactic acid and the acidification of the TME with low pH caused by lactate have an impact on T cells. The research revealed that lactic acid impeded the infiltration of CD8^+^ T cells by promoting the interleukin (IL)-23 expression and secretion (81, 82). Moreover, lactic acid blunted the proliferation, degranulation, motility, and expression of effector molecules (IFN-γ, granzyme, and perforin) (83–85). Mechanically, lactic acid impaired the TCR-triggered phosphorylation of p38 and c-Jun N-terminal kinase/c-Jun in Cytotoxic T lymphocytes, which is involved in IFN-γ production (84). Another study reported that lactic acid prevented the translation of IFN-γ by allowing GAPDH to bind to IFN-γ mRNA (77, 86). Therefore, the CTL function could be restored after treatment with lactate-free medium (83). The acidification of the TME also decreased the IFN-γ production by downregulating the NFAT in T and NK cells, triggering a tumor immune escape (69). Similarly, acidic conditions impaired the antitumor immunity by disturbing the calcineurin-mediated nuclear translocation of NFAT (87). The pH values within the TME mostly decrease between 6.0 and 7.0, but the lowest pH could reach 5.6 (88, 89). The reduced extracellular pH impaired almost all aspects of the CD8^+^ and CD4^+^ lymphocyte function: activation, cytotoxicity, chemotaxis, motility, and proliferation (89–91). Furthermore, lactate and decreased pH showed a synergistic effect on T cells by inducing apoptosis after 24 h and reducing the IFN-γ and IL-2 production (83). Numerous studies have proven that neutralization of the acidic TME with proton pump inhibitors or bicarbonate can restore T-cell function to improve antitumor responses to immunotherapy (91, 92).

In summary, the LDHA-lactate-acidic microenvironment establishes a barrier not only for T-cell numbers but also for T-cell responses. As the initiator, LDHA is a promising target for immunotherapy.

### The effect of LDHA in T cells on T-cell responses

It is instructive to note that aerobic glycolysis is a hallmark of activated T cells, which indicates the intrinsic role of LDHA in T-cell responses (**Figure 3B**). When naive T cells were activated with plate-bound anti-CD3 and anti-CD28, TCR signaling promoted the activation of pyruvate dehydrogenase kinase 1 (PDHK1), suppressing the mitochondrial import of pyruvate (93). Meanwhile, TCR induced the LDHA expression through the phosphoinositide 3-kinase (PI3K)/AKT signaling in activated T cells and then catalyzed the lactate production (94, 95). Indeed, LDHA induced the immature thymocyte antigen-1 (IMT-1) expression from the cytoplasm to the cell surface membrane during the thymocytic differentiation, the process of which is critical for the selection of thymocytes (96). However, LDHA deficiency in CD4^+^ T cells did not affect the thymic development of Treg cells or T-cell homeostasis (97). Furthermore, LDHA deficiency impaired the T-cell activation, proliferation, and migration and the Th17 cell differentiation partly mediated by the defective termination of the Akt-regulated Foxo1-dependent gene expression program (95). LDHA promoted the IFN-γ expression by maintaining high levels of acetyl coenzyme A to enhance the histone acetylation and transcription of IFN-γ but not *via* a 3′-UTR-dependent mechanism of translation *in vivo* (97). In addition to CD4^+^ T cells, LDHA deficiency resulted in a defective CD8^+^ T-cell expansion and differentiation by impairing the Akt and Foxo1 phosphorylation (94). Moreover, LDHA regulated the differentiation of CD8^+^ T-cell effectors into T memory stem cells (Tscm). LDHA inhibition combined with IL-21 *in vitro* promoted the formation of Tscm with increased antitumor activity *in vivo* after adoptive transfer (98).

Taken together, the above evidence indicates that targeting LDHA to modulate the effector functions of T cells in antitumor responses is an efficient strategy for immunotherapy.

## Combining LDHA inhibitors with T cell-mediated immunotherapy

In the light of the important role of LDHA in oncology, selective LDHA inhibition can be deemed as a potentially safe target. To date, significant progress has been achieved in the discovery and development of selective small-molecule LDHA inhibitors. Recently, there are more researchers who reviewed the state of the LDHA inhibitors (99–103). Albeit the inhibitors with a promising antitumor activity both *in vitro* and *in vivo* have been revealed, none of them showed any real clinical benefit. Only one phase III clinical trial of gossypol combined with docetaxel and cisplatin scheme in advanced non-small-cell lung cancer with apurinic/apyrimidinic endonuclease 1 high expression was conducted by the Third Military Medical University (ClinicalTrials.gov Identifier: NCT01977209). The purpose of this study was to find out whether gossypol can improve the sensitivity of the cisplatin-based chemotherapy. However, no study results were posted for this study (Source of information: ClinicalTrials.gov). This is probably due to some reasons: one is that very few clinical applications associated with LDHA inhibition until the relationships between LDHA and aerobic glycolysis were recently discovered. Another reason is that a high serum LDHA is only considered as a robust biomarker of a poor prognosis (103). Meanwhile, the nature of the LDHA structure has not been understood for a long time (102). Moreover, the highly unspecific toxicity or the limited membrane permeability of inhibitors is also a limiting factor (100). Therefore, a progressive increase in the discovery of new LDHA inhibitors with improvement in selectivity, inhibitory activity, low toxicity, and delivery is hopefully accessible in the clinic soon.

Given the role of LDHA in T-cell responses, the combination of LDHA inhibition with T cell-mediated immunotherapy holds promise to patients with tumors. The combination of LDHA depletion with anti-human prostate-specific membrane antigen (hPSMA)-Chimeric antigen receptor T cell therapy could significantly retard tumor growth (104). Moreover, a recent study reported that the shRNA-mediated blockade of LDHA improved the efficacy of anti-PD-1 therapy by enhancing T-cell infiltration in melanoma (70). ML-05 is a novel potent LDHA inhibitor. In a mouse model of B16F10 melanoma, intratumoral injection of ML-05 significantly suppressed tumor growth and released an antitumor immune response of T-cell subsets (Th1 and GMZB^+^CD8 T cells) in the TME. Furthermore, ML-05 treatment combined with anti-PD-1 antibody or stimulator of interferon genes protein (STING) could enhance the antitumor activity in the B16F10 melanoma model (105). Unfortunately, the clinical success of treatment strategies that combine LDHA inhibitor with T cell-mediated immunotherapy is lacking.

## Current challenges and future directions

Cellular metabolic reprogramming, such as aerobic glycolysis, is a marked feature of tumor cells and immune cells in the TME. As an enzyme that catalyzes the final step of glycolysis, LDHA is the focus of research. In this review, we recapitulated the LDHA structure, location, and biological features as well as the epigenetic mechanisms of the LDHA expression. However, the literature regarding how epigenetic modifications regulate LDHA expression is limited. Moreover, most of the data from the above studies were identified in tumor cells, while evidence in other cells such as immune cells is deficient.

Furthermore, we summarized the extrinsic (tumor cells) and intrinsic (T cells) effects of LDHA on T-cell responses to tumors. The LDHA-lactate-acidic microenvironment established a barrier not only for T-cell populations but also for T-cell responses. Moreover, some small-molecule LDHA inhibitors play a marked effect on tumor burden, metastases, and cell death. However, few studies have evaluated the response changes of immune cells in the context of LDHA inhibitors in tumor treatment.

**Figure 4:**
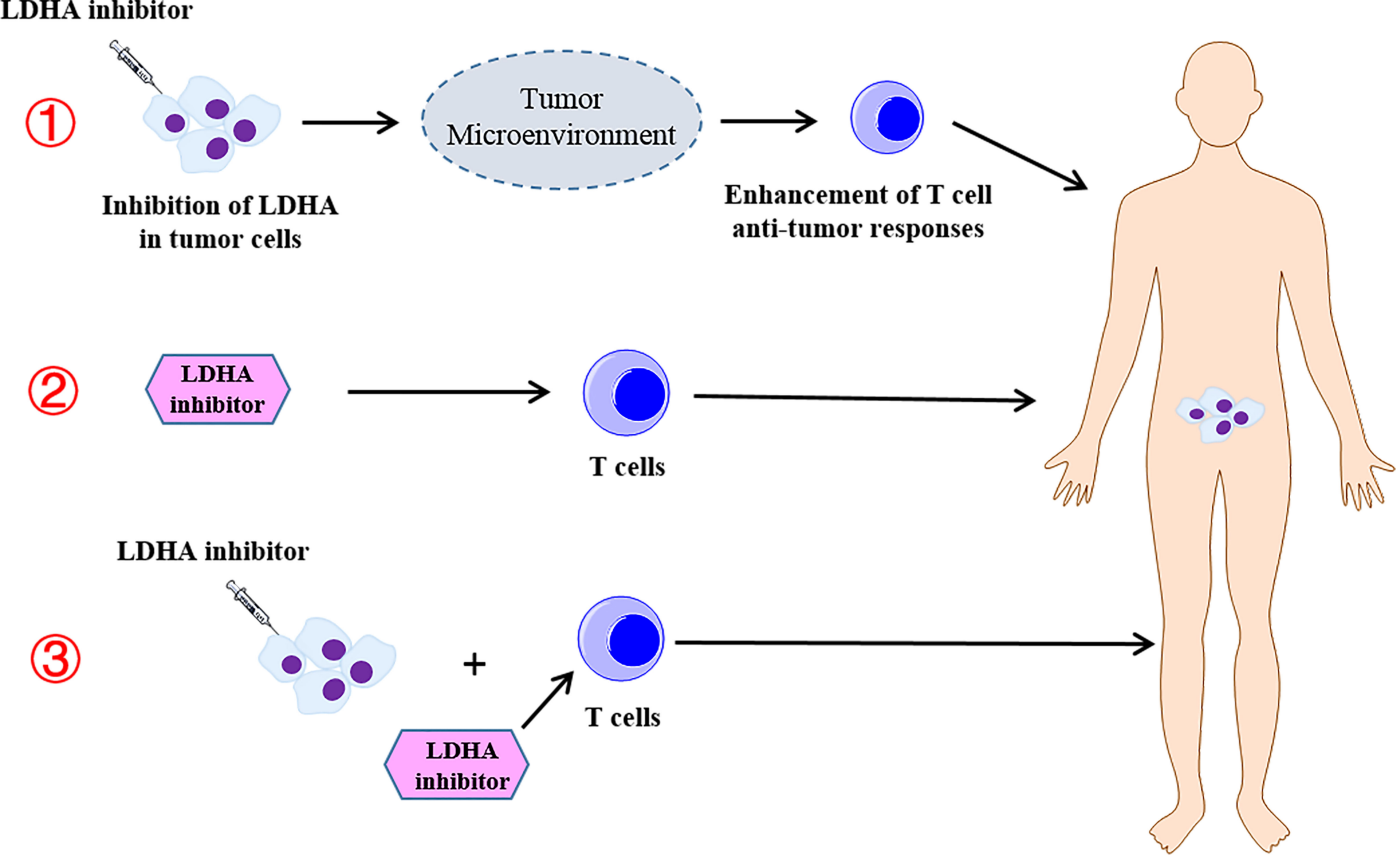
The different strategies of the combination therapy with the lactate dehydrogenase A (LDHA) inhibitor and T-cell immunotherapy. **①** Targeting the inhibition of LDHA in the tumor and regulating the tumor microenvironment to increase the T-cell antitumor response. **②** Targeting LDHA in T cells to enhance the efficacy of the adoptive T-cell therapy. **③** Simultaneous treatment of tumor cells and T cells with LDHA inhibitors to enhance the antitumor efficacy.

In this review, we also summarized the current studies of the combination therapy with LDHA-targeted therapies and T cell-mediated immunotherapy. However, these studies are designed for animal tumor models, and few clinical trials are designed to assess the therapeutic efficacy of combined therapy. Thus, further studies to elucidate the clinical efficiency of the combined therapy will be appreciated. It is worth noting that in the clinical trial of LDHA inhibitors combined with T-cell immunotherapy, a variety of different strategies should be adopted to enhance the efficacy, such as targeting the inhibition of LDHA in tumors and regulating the TME to increase the T-cell antitumor response, targeting LDHA in T cells to enhance the efficacy of ACT, and simultaneous treatment of tumor cells and T cells with LDHA inhibitors to enhance the antitumor efficacy (**Figure 4**).

## Author contributions

YT and SG drafted the original manuscripts. SG provided some constructive comments on the revision of the manuscripts. LZ and WZ reviewed and edited the manuscript. CZ guided on the structure of the manuscript. WZ provided the funding. All authors contributed to the article and approved the submitted version.

## Funding

Jiangsu Elderly Health Research Program (Grant No. LKM2022031).

## Conflict of interest

The authors declare that the research was conducted in the absence of any commercial or financial relationships that could be construed as a potential conflict of interest.

## Publisher’s note

All claims expressed in this article are solely those of the authors and do not necessarily represent those of their affiliated organizations, or those of the publisher, the editors and the reviewers. Any product that may be evaluated in this article, or claim that may be made by its manufacturer, is not guaranteed or endorsed by the publisher.

## References

[1] Yousefi M Bahrami T Salmaninejad A Nosrati R Ghaffari P Ghaffari SH. Lung cancer-associated brain metastasis: Molecular mechanisms and therapeutic options. Cell Oncol (Dordr). (2017) 40(5):419–41. doi: 10.1007/s13402-017-0345-5 PMC13001560 28921309

[2] Abbott M Ustoyev Y. Cancer and the immune system: The history and background of immunotherapy. Semin Oncol Nurs. (2019) 35(5):150923. doi: 10.1016/j.soncn.2019.08.002 31526550

[3] Waldman AD Fritz JM Lenardo MJ. A guide to cancer immunotherapy: from T cell basic science to clinical practice. Nat Rev Immunol (2020) 20(11):651–68. doi: 10.1038/s41577-020-0306-5 PMC7238960 32433532

[4] Giannone G Ghisoni E Genta S Scotto G Tuninetti V Turinetto M. Immuno-metabolism and microenvironment in cancer: Key players for immunotherapy. Int J Mol Sci (2020) 21(12):4414. doi: 10.3390/ijms21124414 32575899 PMC7352562

[5] Leone RD Powell JD. Metabolism of immune cells in cancer. Nat Rev Cancer. (2020) 20(9):516–31. doi: 10.1038/s41568-020-0273-y PMC8041116 32632251

[6] Hanahan D Weinberg RA. Hallmarks of cancer: the next generation. Cell. (2011) 144(5):646–74. doi: 10.1016/j.cell.2011.02.013 21376230

[7] Pascale RM Calvisi DF Simile MM Feo CF Feo F. The warburg effect 97 years after its discovery. Cancers (Basel). (2020) 12(10):2819. doi: 10.3390/cancers12102819 33008042 PMC7599761

[8] Teijeira A Garasa S Etxeberria I Gato-Canas M Melero I Delgoffe GM. Metabolic consequences of T-cell costimulation in anticancer immunity. Cancer Immunol Res (2019) 7(10):1564–9. doi: 10.1158/2326-6066 31575551

[9] Cascone T McKenzie JA Mbofung RM Punt S Wang Z Xu C. Increased tumor glycolysis characterizes immune resistance to adoptive T cell therapy. Cell Metab (2018) 27(5):977–87.e4. doi: 10.1016/j.cmet.2018.02.024 29628419 PMC5932208

[10] Renner K Bruss C Schnell A Koehl G Becker HM Fante M. Restricting glycolysis preserves T cell effector functions and augments checkpoint therapy. Cell Rep (2019) 29(1):135–50.e9. doi: 10.1016/j.celrep.2019.08.068 31577944

[11] Ding J Karp JE Emadi A. Elevated lactate dehydrogenase (LDH) can be a marker of immune suppression in cancer: Interplay between hematologic and solid neoplastic clones and their microenvironments. Cancer biomark (2017) 19(4):353–63. doi: 10.3233/CBM-160336 PMC13020749 28582845

[12] Miao P Sheng S Sun X Liu J Huang G. Lactate dehydrogenase a in cancer: a promising target for diagnosis and therapy. IUBMB Life (2013) 65(11):904–10. doi: 10.1002/iub.1216 24265197

[13] Markert CL SJ Whitt GS. Evolution of a gene. multiple genes for LDH isozymes provide a model of the evolution of gene structure, function and regulation. Science. (1975) 189(4197):102–14. doi: 10.1126/science.1138367 1138367

[14] Everse J KN. Lactate dehydrogenases structure and function. Adv Enzymol Relat Areas Mol Biol (1973) 1973(37):61–133. doi: 10.1002/9780470122822.ch2 4144036

[15] Kayser G KA Sienel W Schulte-Uentrop L Mattern D Aumann K Stickeler E. Lactate-dehydrogenase 5 is overexpressed in non-small cell lung cancer and correlates with the expression of the transketolase-like protein 1. Diagn Pathol (2010) 5:22. doi: 10.1186/1746-1596-5-22 20385008 PMC2861018

[16] Adams MJ MAJ Rossmann MG Schevitz RW Wonacott AJ. The structure of the nicotinamide-adenine dinucleotide coenzyme when bound to lactate dehydrogenase. J Mol Biol (1970) 51(1):31–8. doi: 10.1016/0022-2836(70)90267-6 4320426

[17] Jafary F Ganjalikhany MR Moradi A Hemati M Jafari S. Novel peptide inhibitors for lactate dehydrogenase a (LDHA): A survey to inhibit LDHA activity *via* disruption of protein-protein interaction. Sci Rep (2019) 9(1):4686. doi: 10.1038/s41598-019-38854-7 30886157 PMC6423238

[18] Waldman AD Clarke AR Wigley DB Barstow DA Atkinson T Chia WN. The use of genetically engineered tryptophan to identify the movement of a domain of b. stearothermophilus lactate dehydrogenase with the process which limits the steady-state turnover of the enzyme. Biochem Biophys Res Commun (1988) 150(2):752–9. HK. doi: 10.1016/0006-291X(88)90455-X 3422557

[19] Read JA WV Eszes CM Sessions RB Brady RL. Structural basis for altered activity of m- and h-isozyme forms of human lactate dehydrogenase. Proteins. (2001) 43(2):175–85. doi: 10.1002/1097-0134(20010501)43:2<175::AID-PROT1029>3.0.CO;2-# 11276087

[20] Clarke AR WD Chia WN Barstow D Atkinson T Holbrook JJ. Site-directed mutagenesis reveals role of mobile arginine residue in lactate dehydrogenase catalysis. Nature. (1986) 324(6098):699–702. doi: 10.1038/324699a0 3796734

[21] Dempster S Harper S Moses JE Dreveny I. Structural characterization of the apo form and NADH binary complex of human lactate dehydrogenase. Acta Crystallogr D Biol Crystallogr. (2014) 70(Pt 5):1484–90. doi: 10.1107/S1399004714005422 PMC4014127 24816116

[22] Sun R Li X Li Y Zhang X Li X Li X. Screening of novel inhibitors targeting lactate dehydrogenase a *via* four molecular docking strategies and dynamics simulations. J Mol Model (2015) 21(5):133. doi: 10.1007/s00894-015-2675-4 25934158

[23] Hart KW CA Wigley DB Chia WN Barstow DA Atkinson T Holbrook JJ. The importance of arginine 171 in substrate binding by bacillus stearothermophilus lactate dehydrogenase. Biochem Biophys Res Commun (1987) 146(1):346–53. doi: 10.1016/0006-291X(87)90731-5 3606622

[24] Hart KW CA Wigley DB Waldman AD Chia WN Barstow DA Atkinson T. A strong carboxylate-arginine interaction is important in substrate orientation and recognition in lactate dehydrogenase. Biochim Biophys Acta (1987) 914(3):294–8. doi: 10.1016/0167-4838(87)90289-5 3113484

[25] Peng HL Deng H Dyer RB Callender R. Energy landscape of the michaelis complex of lactate dehydrogenase: relationship to catalytic mechanism. Biochemistry. (2014) 53(11):1849–57. doi: 10.1021/bi500215a PMC3985751 24576110

[26] Qiu L Gulotta M Callender R. Lactate dehydrogenase undergoes a substantial structural change to bind its substrate. Biophys J (2007) 93(5):1677–86. doi: 10.1529/biophysj.107.109397 PMC1948838 17483169

[27] Dunn CR Wilks HM Halsall DJ Atkinson T Clarke AR Muirhead H. Design and synthesis of new enzymes based on the lactate dehydrogenase framework. Philos Trans R Soc Lond B Biol Sci (1991) 332(1263):177–84. doi: 10.1098/rstb.1991.0047 1678537

[28] Kedzierski P MK Clarke AR Holbrook JJ. The A245K mutation exposes another stage of the bacterial l-lactate dehydrogenase reaction mechanism. Biochemistry. (2001) 40(24):7247–52. doi: 10.1021/bi0026775 11401572

[29] Brooks GA DH Brown M Sicurello JP Butz CE. Role of mitochondrial lactate dehydrogenase and lactate oxidation in the intracellular lactate shuttle. Proc Natl Acad Sci U S A. (1999) 96(3):1129–34. doi: 10.1073/pnas.96.3.1129 PMC15362 9927705

[30] Reddy MA SS. Nuclear activation and translocation of mitogen-activated protein kinases modulated by ethanol in embryonic liver cells. Biochim Biophys Acta (2000) 1497(2):271–8. doi: 10.1016/s0167-4889(00)00058-6 10903432

[31] Lenzen S. A fresh view of glycolysis and glucokinase regulation: history and current status. J Biol Chem (2014) 289(18):12189–94. doi: 10.1074/jbc.R114.557314 PMC4007419 24637025

[32] Maekawa M. Lactate dehydrogenase isoenzymes. J Chromatogr (1988) 429:373–98. doi: 10.1016/S0378-4347(00)83879-7 3062027

[33] Liu Y Guo JZ Liu Y Wang K Ding W Wang H. Nuclear lactate dehydrogenase a senses ROS to produce alpha-hydroxybutyrate for HPV-induced cervical tumor growth. Nat Commun (2018) 9(1):4429. doi: 10.1038/s41467-018-06841-7 30356100 PMC6200739

[34] Feng Y Xiong Y Qiao T Li X Jia L Han Y. Lactate dehydrogenase a: A key player in carcinogenesis and potential target in cancer therapy. Cancer Med (2018) 7(12):6124–36. doi: 10.1002/cam4.1820 PMC6308051 30403008

[35] Woodford MR CV Backe SJ Bratslavsky G Mollapour M. Structural and functional regulation of lactate dehydrogenase-a in cancer future med chem. Future Med Chem (2020) 12(5):439–55. doi: 10.4155/fmc-2019-0287 32064930

[36] Zhang Y SZ Jia J Du T Zhang N Tang Y Fang Y. Overview of histone modification. Adv Exp Med Biol (2021) 1283:1–16. doi: 10.1007/978-981-15-8104-5_1 33155134

[37] Su Y Yu QH Wang XY Yu LP Wang ZF Cao YC. JMJD2A promotes the warburg effect and nasopharyngeal carcinoma progression by transactivating LDHA expression. BMC Cancer. (2017) 17(1):477. doi: 10.1186/s12885-017-3473-4 28693517 PMC5504777

[38] Zhong L D'Urso A Toiber D Sebastian C Henry RE Vadysirisack DD. The histone deacetylase Sirt6 regulates glucose homeostasis *via* Hif1alpha. Cell. (2010) 140(2):280–93. doi: 10.1016/j.cell.2009.12.041 PMC2821045 20141841

[39] Shun CT Lin SK Hong CY Lin CF Liu CM. Sirtuin 6 modulates hypoxia-induced autophagy in nasal polyp fibroblasts *via* inhibition of glycolysis. Am J Rhinol Allergy (2016) 30(3):179–85. doi: 10.2500/ajra.2016.30.4282 26803106

[40] Angeloni A Bogdanovic O. Enhancer DNA methylation: implications for gene regulation. Essays Biochem (2019) 63(6):707–15. doi: 10.1042/EBC20190030 31551326

[41] de Bustros A NB Silverman A Ehrlich G Poiesz B Baylin SB. The short arm of chromosome 11 is a hot spot for hypermethylation in human neoplasia. Proc Natl Acad Sci U S A. (1988) 85(15):5693–7. doi: 10.1073/pnas.85.15.5693 PMC281826 2840671

[42] Zhang JJ Shao C Yin YX Sun Q Li YN Zha YW. Hypoxia-related signature is a prognostic biomarker of pancreatic cancer. Dis Markers. (2022) 2022:6449997. doi: 10.1155/2022/6449997 35789607 PMC9250441

[43] Maekawa M IM Sasaki MS Kaneko A Ushiama M Sugano K Takayama J. Electrophoretic variant of a lactate dehydrogenase isoenzyme and selective promoter methylation of the LDHA gene in a human retinoblastoma cell line. Clin Chem (2002) 48(11):1938–45. doi: 10.1093/clinchem/48.11.1938 12406979

[44] Chesnelong C Chaumeil MM Blough MD Al Najjar M Stechishin OD Chan JA. Lactate dehydrogenase a silencing in IDH mutant gliomas. Neuro Oncol (2014) 16(5):686–95. doi: 10.1093/neuonc/not243 PMC3984548 24366912

[45] Ruiz-Rodado V Malta TM Seki T Lita A Dowdy T Celiku O. Metabolic reprogramming associated with aggressiveness occurs in the G-CIMP-high molecular subtypes of IDH1mut lower grade gliomas. Neuro Oncol (2020) 22(4):480–92. doi: 10.1093/neuonc/noz207 PMC7158660 31665443

[46] Givechian KB Garner C Benz S Rabizadeh S Soon-Shiong P. Glycolytic expression in lower-grade glioma reveals an epigenetic association between IDH mutation status and PDL1/2 expression. Neurooncol Adv (2021) 3(1):vdaa162. doi: 10.1093/noajnl/vdaa162 33532725 PMC7837356

[47] Hamadneh L Al-Lakkis L Alhusban AA Tarawneh S Abu-Irmaileh B Albustanji S. Changes in lactate production, lactate dehydrogenase genes expression and DNA methylation in response to tamoxifen resistance development in MCF-7 cell line. Genes (Basel) (2021) 12(5):777. doi: 10.3390/genes12050777 34069745 PMC8160872

[48] Gu J Zhan Y Zhuo L Zhang Q Li G Li Q. Biological functions of m(6)A methyltransferases. Cell Biosci (2021) 11(1):15. doi: 10.1186/s13578-020-00513-0 33431045 PMC7798219

[49] Zhang K Zhang T Yang Y Tu W Huang H Wang Y. N(6)-methyladenosine-mediated LDHA induction potentiates chemoresistance of colorectal cancer cells through metabolic reprogramming. Theranostics. (2022) 12(10):4802–17. doi: 10.7150/thno.73746 PMC9254245 35832094

[50] Correia de Sousa M Gjorgjieva M Dolicka D Sobolewski C Foti M. Deciphering miRNAs' action through miRNA editing. Int J Mol Sci (2019) 20(24):6249. doi: 10.3390/ijms20246249 31835747 PMC6941098

[51] Wang J WH Liu A Fang C Hao J Wang Z. Lactate dehydrogenase a negatively regulated by miRNAs promotes aerobic glycolysis and is increased in colorectal cancer. Oncotarget. (2015) 6(23):19456–68. doi: 10.18632/oncotarget.3318 PMC4637298 26062441

[52] Khordadmehr M Jigari-Asl F Ezzati H Shahbazi R Sadreddini S Safaei S. A comprehensive review on miR-451: A promising cancer biomarker with therapeutic potential. J Cell Physiol (2019) 234(12):21716–31. doi: 10.1002/jcp.28888 31140618

[53] Li L Liu H Du L Xi P Wang Q Li Y. miR-449a suppresses LDHA-mediated glycolysis to enhance the sensitivity of non-small cell lung cancer cells to ionizing radiation. Oncol Res (2018) 26(4):547–56. doi: 10.3727/096504017X15016337254605 PMC7844793 28800787

[54] He Y Chen X Yu Y Li J Hu Q Xue C. LDHA is a direct target of miR-30d-5p and contributes to aggressive progression of gallbladder carcinoma. Mol Carcinog. (2018) 57(6):772–83. doi: 10.1002/mc.22799 29569755

[55] Tian Y CY Han AL. MiR-1271 inhibits cell proliferation and metastasis by targeting LDHA in endometrial cance. Eur Rev Med Pharmacol Sci (2019) 23(13):5648–56. doi: 10.26355/eurrev_201907_18300 31298316

[56] Hua S Liu C Liu L Wu D. miR-142-3p inhibits aerobic glycolysis and cell proliferation in hepatocellular carcinoma *via* targeting LDHA. Biochem Biophys Res Commun (2018) 496(3):947–54. doi: 10.1016/j.bbrc.2018.01.112 29360449

[57] Han RL Wang FP Zhang PA Zhou XY Li Y. miR-383 inhibits ovarian cancer cell proliferation, invasion and aerobic glycolysis by targeting LDHA. Neoplasma. (2017) 64(2):244–52. doi: 10.4149/neo_2017_211 28043152

[58] Chow TF Youssef YM Lianidou E Romaschin AD Honey RJ Stewart R. Differential expression profiling of microRNAs and their potential involvement in renal cell carcinoma pathogenesis. Clin Biochem (2010) 43(1-2):150–8. doi: 10.1016/j.clinbiochem.2009.07.020 19646430

[59] Ghosh AK Shanafelt TD Cimmino A Taccioli C Volinia S Liu CG. Aberrant regulation of pVHL levels by microRNA promotes the HIF/VEGF axis in CLL b cells. Blood. (2009) 113(22):5568–74. doi: 10.1182/blood-2008-10-185686 PMC2689054 19336759

[60] Shangguan H Feng H Lv D Wang J Tian T Wang X. Circular RNA circSLC25A16 contributes to the glycolysis of non-small-cell lung cancer through epigenetic modification. Cell Death Dis (2020) 11(6):437. doi: 10.1038/s41419-020-2635-5 32513983 PMC7280231

[61] Hu R Chen S Yan J. Blocking circ-CNST suppresses malignant behaviors of osteosarcoma cells and inhibits glycolysis through circ-CNST-miR-578-LDHA/PDK1 ceRNA networks. J Orthopaedic Surg Res (2021) 16(1):300. doi: 10.1186/s13018-021-02427-0 PMC8103765 33962616

[62] Zhang Q Wang L Cao L Wei T. Novel circular RNA circATRNL1 accelerates the osteosarcoma aerobic glycolysis through targeting miR-409-3p/LDHA. Bioengineered. (2021) 12(2):9965–75. doi: 10.1080/21655979.2021.1985343 PMC8809938 34635009

[63] Guan H Luo W Liu Y Li M. Novel circular RNA circSLIT2 facilitates the aerobic glycolysis of pancreatic ductal adenocarcinoma *via* miR-510-5p/c-Myc/LDHA axis. Cell Death Disease. (2021) 12(7):645. doi: 10.1038/s41419-021-03918-y 34168116 PMC8225611

[64] Xing Z Wang R Wang X Liu J Zhang M Feng K. CircRNA circ-PDCD11 promotes triple-negative breast cancer progression *via* enhancing aerobic glycolysis. Cell Death Discovery (2021) 7(1):218. doi: 10.1038/s41420-021-00604-y 34420029 PMC8380247

[65] Lu X Chen L Li Y Huang R Meng X Sun F. Long non-coding RNA LINC01207 promotes cell proliferation and migration but suppresses apoptosis and autophagy in oral squamous cell carcinoma by the microRNA-1301-3p/lactate dehydrogenase isoform a axis. Bioengineered. (2021) 12(1):7780–93. doi: 10.1080/21655979.2021.1972784 PMC8806684 34463208

[66] Hu J Huang L Ding Q Lv J Chen Z. Long noncoding RNA HAGLR sponges miR-338-3p to promote 5-fu resistance in gastric cancer through targeting the LDHA-glycolysis pathway. Cell Biol Int (2022) 46(2):173–84. doi: 10.1002/cbin.11714 PMC9300138 34658120

[67] Ni S Liu Y Zhong J Shen Y. Inhibition of LncRNA-NEAT1 alleviates intestinal epithelial cells (IECs) dysfunction in ulcerative colitis by maintaining the homeostasis of the glucose metabolism through the miR-410-3p-LDHA axis. Bioengineered. (2022) 13(4):8961–71. doi: 10.1080/21655979.2022.2037957 PMC9161899 35735114

[68] Van Wilpe S Koornstra R Den Brok M De Groot JW Blank C De Vries J. Lactate dehydrogenase: a marker of diminished antitumor immunity. Oncoimmunology. (2020) 9(1):1731942. doi: 10.1080/2162402X.2020.1731942 32158624 PMC7051189

[69] Brand A Singer K Koehl GE Kolitzus M Schoenhammer G Thiel A. LDHA-associated lactic acid production blunts tumor immunosurveillance by T and NK cells. Cell Metab (2016) 24(5):657–71. doi: 10.1016/j.cmet.2016.08.011 27641098

[70] Daneshmandi S Wegiel B Seth P. Blockade of lactate dehydrogenase-a (LDH-a) improves efficacy of anti-programmed cell death-1 (PD-1) therapy in melanoma. Cancers (Basel). (2019) 11(4):450. doi: 10.3390/cancers11040450 30934955 PMC6521327

[71] Serganova I Cohen IJ Vemuri K Shindo M Maeda M Mane M. LDH-a regulates the tumor microenvironment *via* HIF-signaling and modulates the immune response. PLoS One (2018) 13(9):e0203965. doi: 10.1371/journal.pone.0203965 30248111 PMC6153000

[72] Singer K Kastenberger M Gottfried E Hammerschmied CG Buttner M Aigner M. Warburg phenotype in renal cell carcinoma: high expression of glucose-transporter 1 (GLUT-1) correlates with low CD8(+) T-cell infiltration in the tumor. Int J Cancer. (2011) 128(9):2085–95. doi: 10.1002/ijc.25543 20607826

[73] de la Cruz-Lopez KG Castro-Munoz LJ Reyes-Hernandez DO Garcia-Carranca A Manzo-Merino J. Lactate in the regulation of tumor microenvironment and therapeutic approaches. Front Oncol (2019) 9:1143. doi: 10.3389/fonc.2019.01143 31737570 PMC6839026

[74] Angelin A Gil-de-Gomez L Dahiya S Jiao J Guo L Levine MH. Foxp3 reprograms T cell metabolism to function in low-glucose, high-lactate environments. Cell Metab (2017) 25(6):1282–93.e7. doi: 10.1016/j.cmet.2016.12.018 28416194 PMC5462872

[75] Comito G Iscaro A Bacci M Morandi A Ippolito L Parri M. Lactate modulates CD4(+) T-cell polarization and induces an immunosuppressive environment, which sustains prostate carcinoma progression *via* TLR8/miR21 axis. Oncogene. (2019) 38(19):3681–95. doi: 10.1038/s41388-019-0688-7 30664688

[76] Kumagai S Koyama S Itahashi K Tanegashima T Lin YT Togashi Y. Lactic acid promotes PD-1 expression in regulatory T cells in highly glycolytic tumor microenvironments. Cancer Cell (2022) 40(2):201–18.e9. doi: 10.1016/j.ccell.2022.01.001 35090594

[77] Haas R Smith J Rocher-Ros V Nadkarni S Montero Melendez T D'Acquisto F. Lactate regulates metabolic and pro-inflammatory circuits in control of T cell migration and effector functions. PLoS Biol (2015) 13(7):e1002202. doi: 10.1371/journal.pbio.1002202 26181372 PMC4504715

[78] Chen Y FZ Kuang X Zhao P Chen B Fang Q Cheng W. Increased lactate in AML blasts upregulates TOX expression, leading to exhaustion of CD8 + cytolytic T cells. Am J Cancer Res (2021) 11(11):5726–42. PMC8640829 34873490

[79] Feng Q Liu Z Yu X Huang T Chen J Wang J. Lactate increases stemness of CD8 + T cells to augment anti-tumor immunity. Nat Commun (2022) 13(1):4981. doi: 10.1038/s41467-022-32521-8 36068198 PMC9448806

[80] Wen J Cheng S Zhang Y Wang R Xu J Ling Z. Lactate anions participate in T cell cytokine production and function. Sci China Life Sci (2021) 64(11):1895–905. doi: 10.1007/s11427-020-1887-7 33580429

[81] Shime H Yabu M Akazawa T Kodama K Matsumoto M Seya T. Tumor-secreted lactic acid promotes IL-23/IL-17 proinflammatory pathway. J Immunol (2008) 180(11):7175–83. doi: 10.4049/jimmunol.180.11.7175 18490716

[82] Langowski JL Zhang X Wu L Mattson JD Chen T Smith K. IL-23 promotes tumour incidence and growth. Nature. (2006) 442(7101):461–5. doi: 10.1038/nature04808 16688182

[83] Fischer K Hoffmann P Voelkl S Meidenbauer N Ammer J Edinger M. Inhibitory effect of tumor cell-derived lactic acid on human T cells. Blood. (2007) 109(9):3812–9. doi: 10.1182/blood-2006-07-035972 17255361

[84] Mendler AN Hu B Prinz PU Kreutz M Gottfried E Noessner E. Tumor lactic acidosis suppresses CTL function by inhibition of p38 and JNK/c-jun activation. Int J Cancer. (2012) 131(3):633–40. doi: 10.1002/ijc.26410 21898391

[85] Fischbeck AJ Ruehland S Ettinger A Paetzold K Masouris I Noessner E. Tumor lactic acidosis: Protecting tumor by inhibiting cytotoxic activity through motility arrest and bioenergetic silencing. Front Oncol (2020) 10:589434. doi: 10.3389/fonc.2020.589434 33364193 PMC7753121

[86] Chang CH Curtis JD Maggi LB Jr. Faubert B Villarino AV O'Sullivan D. Posttranscriptional control of T cell effector function by aerobic glycolysis. Cell. (2013) 153(6):1239–51. doi: 10.1016/j.cell.2013.05.016 PMC3804311 23746840

[87] Hisamitsu T Nakamura TY Wakabayashi S. Na(+)/H(+) exchanger 1 directly binds to calcineurin a and activates downstream NFAT signaling, leading to cardiomyocyte hypertrophy. Mol Cell Biol (2012) 32(16):3265–80. doi: 10.1128/MCB.00145-12 PMC3434548 22688515

[88] Boedtkjer E Pedersen SF. The acidic tumor microenvironment as a driver of cancer. Annu Rev Physiol (2020) 82:103–26. doi: 10.1146/annurev-physiol-021119-034627 31730395

[89] Erra Diaz F Dantas E Geffner J. Unravelling the interplay between extracellular acidosis and immune cells. Mediators Inflamm (2018) 2018:1218297. 30692870 10.1155/2018/1218297 PMC6332927

[90] Nakagawa Y Negishi Y Shimizu M Takahashi M Ichikawa M Takahashi H. Effects of extracellular pH and hypoxia on the function and development of antigen-specific cytotoxic T lymphocytes. Immunol Lett (2015) 167(2):72–86. doi: 10.1016/j.imlet.2015.07.003 26209187

[91] Balgi AD Diering GH Donohue E Lam KK Fonseca BD Zimmerman C. Regulation of mTORC1 signaling by pH. PLoS One (2011) 6(6):e21549. doi: 10.1371/journal.pone.0021549 21738705 PMC3126813

[92] Ippolito L Morandi A Giannoni E Chiarugi P. Lactate: A metabolic driver in the tumour landscape. Trends Biochem Sci (2019) 44(2):153–66. doi: 10.1016/j.tibs.2018.10.011 30473428

[93] Menk AV Scharping NE Moreci RS Zeng X Guy C Salvatore S. Early TCR signaling induces rapid aerobic glycolysis enabling distinct acute T cell effector functions. Cell Rep (2018) 22(6):1509–21. doi: 10.1016/j.celrep.2018.01.040 PMC5973810 29425506

[94] Xu K YN Peng M Stamatiades EG Shyu A Li P Zhang X. Glycolysis fuels phosphoinositide 3-kinase signaling to bolster T cell immunity. Science. (2021) 371(6527):405–10. doi: 10.1126/science.abb2683 PMC8380312 33479154

[95] Xu K Yin N Peng M Stamatiades EG Chhangawala S Shyu A. Glycolytic ATP fuels phosphoinositide 3-kinase signaling to support effector T helper 17 cell responses. Immunity. (2021) 54(5):976–87.e7. doi: 10.1016/j.immuni.2021.04.008 33979589 PMC8130647

[96] Fujishiro Y KH Matsuda T Fuse H Muraguchi A. Lactate dehydrogenase a-dependent surface expression of immature thymocyte antigen-1 an implication for a novel trafficking function of lactate dehydrogenase-a during T cell development. Eur J Immunol (2000) 30(2):516–24. doi: 10.1002/1521-4141(200002)30:2<516::AID-IMMU516>3.0.CO;2-P 10671207

[97] Peng M YN Chhangawala S Xu K Leslie CS Li MO. Aerobic glycolysis promotes T helper 1 cell differentiation through an epigenetic mechanism. Science. (2016) 354(6311):481–4. doi: 10.1126/science.aaf6284 PMC5539971 27708054

[98] Hermans D Gautam S Garcia Canaveras JC Gromer D Mitra S Spolski R. Lactate dehydrogenase inhibition synergizes with IL-21 to promote CD8(+) T cell stemness and antitumor immunity. Proc Natl Acad Sci U S A. (2020) 117(11):6047–55. doi: 10.1073/pnas.1920413117 PMC7084161 32123114

[99] Fiume L MM Vettraino M Di Stefano G. Inhibition of lactate dehydrogenase activity as an approach to cancer therapy. Future Med Chem (2014) 6(4):429–45. doi: 10.4155/fmc.13.206 24635523

[100] Rani R Kumar V. Recent update on human lactate dehydrogenase enzyme 5 (hLDH5) inhibitors: A promising approach for cancer chemotherapy. J Med Chem (2016) 59(2):487–96. doi: 10.1021/acs.jmedchem.5b00168 26340601

[101] Zhang SL He Y Tam KY. Targeting cancer metabolism to develop human lactate dehydrogenase (hLDH)5 inhibitors. Drug Discovery Today (2018) 23(7):1407–15. doi: 10.1016/j.drudis.2018.05.014 29750903

[102] Granchi C PI Rani R Minutolo F. Small-molecule inhibitors of human LDH5. Future Med Chem (2013) 5(16):1967–91. doi: 10.4155/fmc.13.151 PMC3952072 24175747

[103] Claps G Faouzi S Quidville V Chehade F Shen S Vagner S. The multiple roles of LDH in cancer. Nat Rev Clin Oncol (2022) 19:749–762. doi: 10.1038/s41571-022-00686-2 36207413

[104] Mane MM Cohen IJ Ackerstaff E Shalaby K Ijoma JN Ko M. Lactate dehydrogenase a depletion alters MyC-CaP tumor metabolism, microenvironment, and CAR T cell therapy. Mol Ther Oncolytics. (2020) 18:382–95. doi: 10.1016/j.omto.2020.07.006 PMC7452096 32913888

[105] Du M Yu T Zhan Q Li H Zou Y Geng M. Development of a novel lactate dehydrogenase a inhibitor with potent antitumor activity and immune activation. Cancer Sci (2022). doi: 10.1111/cas.15468 PMC9459323 35722994

